# Depressive disorders after cervical spine injuries in amateur athletes

**DOI:** 10.1192/j.eurpsy.2025.1375

**Published:** 2025-08-26

**Authors:** ?. Syrmos

**Affiliations:** 1 Aristotle University, Thessaloniki, Greece

## Abstract

**Introduction:**

Cervical spine injuries are serious traumatic situations with negative effects in the overall health and also in sports performace and health

**Objectives:**

Aim of this study is to present cases of deppresive disorders after cervical spine injurie in amateur athletes

**Methods:**

12 cases are presented. Range of age between 30 and 40 years old. All of them reported depressive disorders during the post traumatic period after cervical spine injuries mainly during amateur athletic activities

**Results:**

All of them they receive appropriate neurological, psychiatric, psycological and rehabilitation support and treatment. They managed to have a good outcome after 24 months follow up.

**Conclusions:**

The development of depressive disorders after such traumatic events remains a strong predictor of a variety of difunctions (social, personal, work etc). The emergence of depressive disorders in many cases remains unexplored and poorly understood. The effect into the the overall health remains a very important factor to investigate. The combination and collaboration of the various medical disciplines is essential in order to help young people.

**Disclosure of Interest:**

None Declared

